# Discovering unknown response patterns in progress test data to improve the estimation of student performance

**DOI:** 10.1186/s12909-023-04172-w

**Published:** 2023-03-29

**Authors:** Miriam Sieg, Iván Roselló Atanet, Mihaela Todorova Tomova, Uwe Schoeneberg, Victoria Sehy, Patrick Mäder, Maren März

**Affiliations:** 1 Charité - Universitätsmedizin Berlin, corporate member of Freie Universität Berlin and Humboldt Universität zu Berlin, AG Progress Test Medizin, Charitéplatz 1, 10117 Berlin, Germany; 2 Charité - Universitätsmedizin Berlin, corporate member of Freie Universität Berlin and Humboldt Universität zu Berlin, Institute of Biometry and Clinical Epidemiology, Charitéplatz 1, 10117 Berlin, Germany; 3 Fakultät für Informatik und Automatisierung, Data-Intensive Systems and Visualization Group (dAI.SY), Technische Universität Ilmenau, Ehrenbergstraße 29, 98693 Ilmenau, Germany; 4 Fakultät für Biowissenschaften, Friedrich Schiller Universität Jena, Schloßgasse 10, 07743 Jena, Germany

**Keywords:** Progress test, Unsupervised machine learning, Supervised machine learning, Student groups, Clustering, k-means, Classification, Ensemble learning, Boosting algorithm, Explainer

## Abstract

**Background:**

The Progress Test Medizin (PTM) is a 200-question formative test that is administered to approximately 11,000 students at medical universities (Germany, Austria, Switzerland) each term. Students receive feedback on their knowledge (development) mostly in comparison to their own cohort. In this study, we use the data of the PTM to find groups with similar response patterns.

**Methods:**

We performed k-means clustering with a dataset of 5,444 students, selected cluster number k = 5, and answers as features. Subsequently, the data was passed to XGBoost with the cluster assignment as target enabling the identification of cluster-relevant questions for each cluster with SHAP. Clusters were examined by total scores, response patterns, and confidence level. Relevant questions were evaluated for difficulty index, discriminatory index, and competence levels.

**Results:**

Three of the five clusters can be seen as “performance” clusters: cluster 0 (*n *= 761) consisted predominantly of students close to graduation. Relevant questions tend to be difficult, but students answered confidently and correctly. Students in cluster 1 (*n* = 1,357) were advanced, cluster 3 (*n* = 1,453) consisted mainly of beginners. Relevant questions for these clusters were rather easy. The number of guessed answers increased. There were two “drop-out” clusters: students in cluster 2 (*n* = 384) dropped out of the test about halfway through after initially performing well; cluster 4 (*n* = 1,489) included students from the first semesters as well as “non-serious” students both with mostly incorrect guesses or no answers.

**Conclusion:**

Clusters placed performance in the context of participating universities. Relevant questions served as good cluster separators and further supported our “performance” cluster groupings.

**Supplementary Information:**

The online version contains supplementary material available at 10.1186/s12909-023-04172-w.

## Background

Progress Testing is a cross-sectional and longitudinal assessment that provides a distinctive and verifiable measure of student knowledge growth and effectiveness ([1] and references therein). Students take the test periodically throughout their studies. Compilation of the test follows a fixed content blueprint, with graduate-level questions resulting in different but comparable tests [2, 3]. The longitudinal nature of the test enables monitoring a student’s progress through to graduation. The cross-sectional nature allows for comparison of students within the same cohort and across cohorts or universities, as the test is identical for all students. Thus, progress tests are a rich source of feedback for individuals, cohorts, and universities [4–7].

In Germany, the ‘Progress Test Medizin’ (PTM) in medical education was jointly introduced by Charité - Universitätsmedizin Berlin (Charité) and Witten/Herdecke University in 1999. Today, the PTM consortium administers a progress test consisting of 200 multiple-choice questions each term to approximately 11,000 students from 17 universities in Germany, Austria, and Switzerland. The PTM is based on a two-dimensional blueprint that maps each question to an organ system and a medical subject [8]. Students answer the questions based on acquired knowledge and motivation. They have 180 min to complete the test and may skip questions. Starting 2018, about half of the participating universities have added “certainty” (or “confidence”) rating to their exam environments. Students indicate their confidence in their answers on a 3-point Likert scale (“I am very sure”, “I am fairly sure”, “I am guessing”) [9] henceforth referred to as confidence level.

The PTM is a formative assessment in which low test-taking effort does not usually result in consequences. However, low test-taking effort may lead to an underestimation of student performance and proficiency, which in turn may lead to negatively biased overall scores that may compromise the validity of the test results [10, 11]. A set of criteria based on the work of Schüttpelz-Brauns et al. [12] and Karay et al. [13] is applied to identify test scores that are due to “low test-taking effort”, as these should be distinguished from those that are due to “insufficient achieved knowledge”.

Students receive detailed, individualized feedback that reflects their current knowledge and knowledge gains for each organ system and medical subject. This feedback is based on numerical scores compared to individual cohorts to account for differences in curriculum. Additional feedback relates one's performance to the knowledge of all students across terms, academic semesters, and universities [1, 4, 14].

A total score does not indicate which cohort a student belongs to or which academic semester they are in, as the increase in knowledge is gradual and not incremental. In addition, total scores cannot be used to infer question-level responses: scores from questions of different content with different confidence levels and difficulty indices may add up to the same total score. Surveys on PTM have shown that students wish to compare their performance to that of other participating universities [15, 16]. We aim to identify groups of students by inferring response patterns using only the response status, correctness of, and confidence in all answers, excluding pre-defined criteria such as test-taking effort (“seriousness”), cohorts, and curricular differences across universities. Educational data mining approaches have been shown to help identify underlying structures in educational data [17] and predict student performance ([18] and references therein). Wang et al. (2021) applied a Markov chain model to identify latent states in the longitudinal trajectories of medical students from one medical school [19].

Since we want our identified groups to be independent from cohorts, clustering is an obvious option. Clustering algorithms identify groups aka clusters, whose objects are more similar to each other than to objects in other clusters. Moreover, they provide better insight into complex data [20]. Clustering has been used to group students according to their proficiency level, mainly to support their learning [21, 22].

We then aim to identify those PTM questions that had the greatest impact on the clustering. We examine those in more detail in terms of intended competence level, difficulty index, and discrimination index in order to increase our understanding of the identified clusters. Hence, we classify the data using the clusters as targets. We use an iterative boosting algorithm that combines a set of simpler models, each with limited predictive power (“weak learners”) [23, 24]. Here, misclassified input data is (mostly) weighted higher in subsequent iterations to promote learning of the algorithm. The overall result is more accurate than each weak learner alone [25, 26]. For example, gradient boosting trees have been used in the analysis of massive open online courses to identify the most important features for either success or failure of students ([27] and references therein). Classification is followed by the explainer algorithm *SHapley Additive Explanation* (SHAP) [28], which allows the derivation of the importance of features per class.

In summary, our goal is to identify unknown underlying patterns using the correctness of and confidence in the answers to the PTM questions, disregarding a student’s university or academic semester, as well as the student’s presumed seriousness of participation. To achieve this, we apply clustering followed by classification and explanation to obtain both new information about differentially performing groups and relevant PTM questions that distinguish the groups.

## Materials and methods

**Fig. 1:**
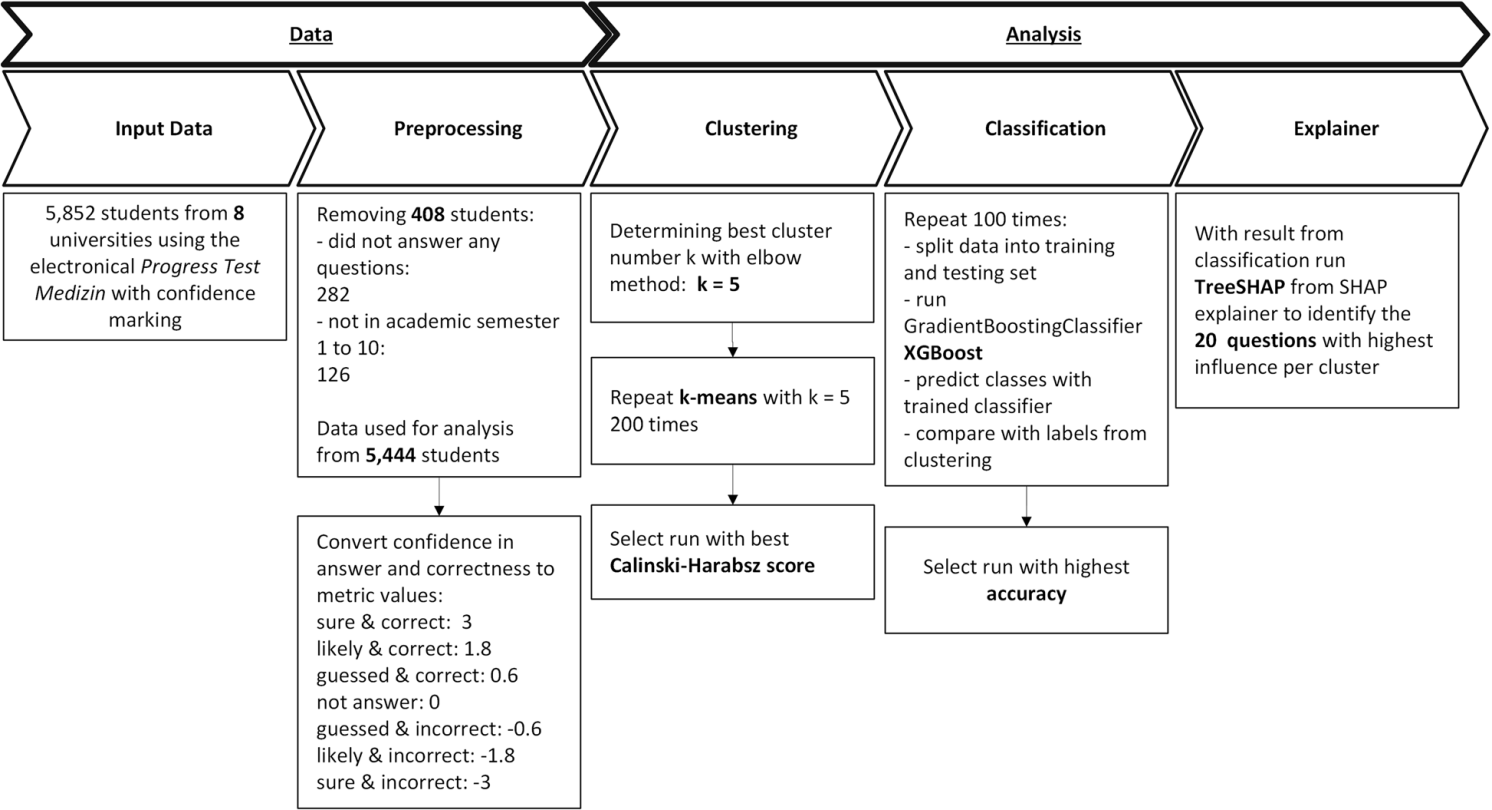
Pipeline flowchart. This flowchart shows the data preprocessing and analysis steps with quality control measures that were followed in this study

In this section, we introduce the pipeline we followed to identify groups aka clusters based on test responses.  Figure 1 provides an overview of the analysis workflow.

### Data pipeline

#### Input data

Eight universities of the PTM consortium using confidence rating agreed to participate in this study. PTM data from the winter term 2020 were used, comprising 5,852 PTM tests from students across eleven academic semesters. Students were anonymized, universities were pseudonymized. The “seriousness” of each participation was obtained from the general analysis of said data. Appendix Fig. 1 shows the percentage of correct answers per student grouped by semester.

The PTM data held the graded answers to each of the 200 questions ordered by their appearance in the test (columns) for each student (rows). Grades were composed of correctness and confidence. Questions were answered either correctly, incorrectly, or not at all. Students indicated their confidence level in each of their answers as “I am very sure” (short: “sure”), “I am fairly sure” (“likely”), or “I am guessing” (“guessed”) [9].

Each question had a difficulty index and discrimination index. Both were test-specific. The percentage of students who answered the question correctly yields the difficulty index (correct [%]) [29, 30]. The higher the difficulty index, the easier the question. The discrimination index (point biserial correlation [31]) indicates how well a question discriminates a high scoring student from a low scoring student. The discrimination index ranges from -1 to 1 [30]. A well discriminating question usually has a discrimination index greater than 0.3 [32].

A field expert assigned each question to one of two competence levels (“apply”, “recall”). “Apply” questions include a brief clinical or laboratory vignette to be interpreted or analyzed, whereas “recall” questions test the student's knowledge of a topic [29].

#### Preprocessing

Only students from academic semesters one to ten who answered at least one question were included in the final dataset. Students in their eleventh academic semester were not included because their participation is voluntary and most of the participating universities do not offer the PTM beyond the tenth academic semester.

For clustering, confidence and correctness had to be converted into metric values. We assigned an initial score of 3 points for “sure” answers, 2 points for “likely” answers, and 1 point for “guessed” answers, with positive scores for correct answers and negative scores for incorrect answers. We then adjusted the scoring to maintain the relative distances of the mean percent correct (difficulty index) for each confidence level (Appendix Table 3). Hence, “sure” answers were scored + (correct) or - (incorrect) 3 points, “likely” answers were scored + or -1.8, and “guessed” answers were scored + or -0.6; unanswered questions were scored 0. The total score for a student was calculated by summing the individual scores for all 200 questions. Hence, the total score had a possible range from -600 to 600.

### Analysis pipeline

#### Algorithms

In our analysis setting, each test per student was an observation, featuring the metrics of scored confidence and correctness of each question. The pipeline was implemented in Python (version 3.8.3 [33]) and consisted of three algorithms: First, we used clustering to detect the underlying patterns. Second, we trained a classifier on the clustered data. Third, we applied an explainer algorithm on these results to extract the relevant features, i.e., questions which distinguished each cluster from the others. The resulting accuracy of the classifier was also used as an evaluation parameter for the clustering algorithm.

##### *Clustering*

We used the *k-means* to cluster our data. *K-means* tries to partition the dataset into *k* distinct non-overlapping clusters. It assigns observations to a cluster such that the Euclidean distance between the observations and the cluster’s centroid is at a minimum [34]. An optimal number of clusters *k* leads to stable, meaningful and interpretable clusters. Interpretability and meaningfulness can decrease with too few, but also with too many clusters [35]. We determined the number of clusters using the elbow method with the Euclidean distance as the distortion score. The *KElbowVisualizer* function from the *Yellowbrick* package [36] returns the optimal cluster number *k* for an explored range of potential cluster numbers. We evaluated the clusters based on the returned *k* for interpretability before deciding on the final *k*. Since *k-means* is known to converge to a local minimum [37], we ran *k-means* 200 times with our final *k*. We then selected the run with the best Calinski-Harabasz score (CHS). The CHS represents the ratio of within-cluster to between-cluster dispersion. The higher the score, the better the cluster separation [38]. Additionally, the accuracy of the classifier further down the pipeline served as an additional performance measure (see below).

##### *Classification*

We performed multiclass classification [39]. Input for classifiers were features and targets. Here, the features were the same as the input for the clustering algorithm; the targets were the clusters assigned by *k-means*. The dataset was split into a training dataset with 75% of the data and a testing dataset with 25% of the data using sklearn [39]. The Gradient Boosting classifier *XGBoost* [40] was used for classification. The default *gbtree* served as the booster and *multi:softprob* as the learning objective for predicting each data point belonging to each class. The learning rate was set to 0.2 and early stopping was selected to avoid overfitting. All other parameters were left at default. We used *mlogloss*, which returns the logistic loss in a multiclass dataset [39, 41] as evaluation metric. The performance of the trained model on the testing dataset was evaluated using the overall accuracy as the performance metric. This step was repeated 100 times with random train-test-splits. The results from the run with the highest accuracy served as input for the explainer.

##### *Explainer*

We used the *TreeSHAP* method of the *SHAP* library to estimate the features’ relevance [42]. The average of the absolute SHAP values represents the global importance of each feature for each class (here: each of our clusters) [43]. For each cluster, we selected the 20 questions with the highest absolute SHAP value as the 20 most relevant questions. We related these questions to their position in the test, their difficulty index, their discrimination index, and their mapping to the intended competence.

## Results

### Preprocessed dataset

We removed 408 students who did not meet our inclusion criteria during preprocessing. Thus, our final dataset included 5,444 students from eight universities. The number of students per academic semester ranged from *N* = 383 in semester 6 to *N* = 942 in semester 3 (Appendix Table 1). Most of the students (*N* = 3,077) came from the same university, while the number of students from the other seven universities ranged from *N* = 152 to *N* = 526 (Appendix Table 2).

Of the 200 questions provided, 110 were of competence “recall” and 90 were of competence “apply”. The mean ± standard deviation of the difficulty index and the discrimination index were 34.08 ± 17.12 and 0.42 ± 0.12, respectively. A discrimination index greater than 0.3 was obtained for 165 questions (histogram of all discrimination indices: Appendix Fig. 2).

**Fig. 2:**
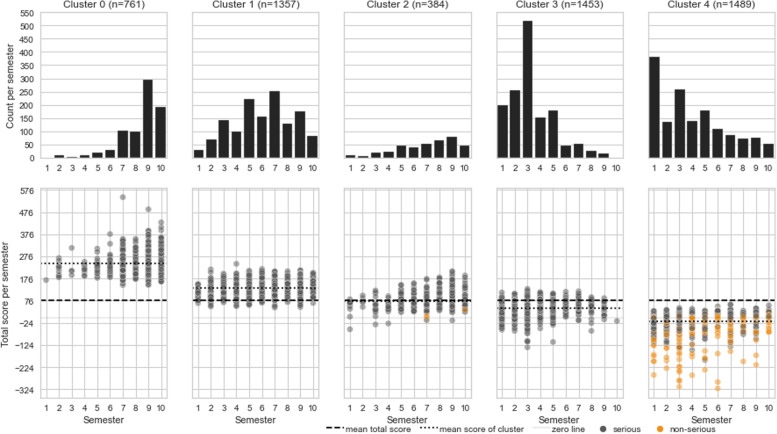
Distribution of academic semesters per cluster and mean total score per cluster. Clusters are sorted from left to right in descending order by the mean total score. Each column shows general overviews for each cluster by academic semester. While the upper bar plots show the number of students, the lower scatterplots show the total scores. Each point in the scatterplot is the total score of one student. Gray points are participations considered serious; orange points are participations considered non-serious

### Clustering

Based on the elbow method, the optimal number of clusters was *k* = 5 (Appendix Fig. 3). With this cluster size, *k-means* was run 200 times (descriptive statistics: Appendix Table 4). CHS differed by only 2.5 with median of 215.04. We selected the best run with a CHS of 215.08. The corresponding cluster assignments were chosen for further analysis.

**Fig. 3:**
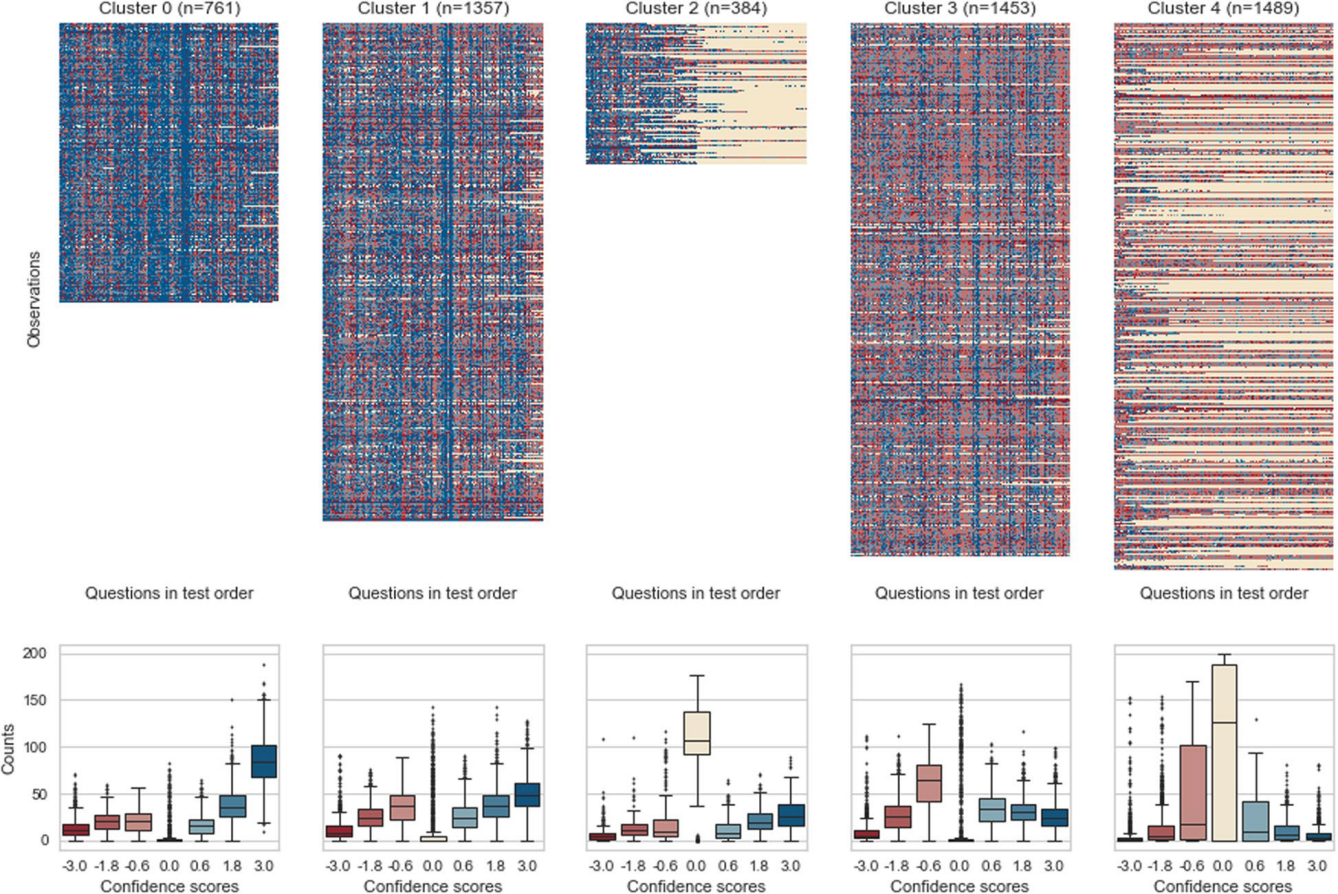
Distribution of confidence and correctness per cluster. Blue colors represent correctly answered questions, red colors represent incorrectly answered questions. The shades of color indicate the level of confidence the student had in their answer. Beige represents unanswered questions. The upper plots are heat maps showing the scores for each answer for each student in the respective cluster (y-axis) ordered from left to right by the position of the question in the test (x-axis). The boxplots in the lower plots show how often a student answered with what confidence and correctness for each cluster. Thus, each boxplot includes all participating students in that cluster

All clusters contained students from all ten academic semesters. However, each cluster showed a tendency to peak at a certain academic semester. Three of the clusters were characterized by different levels of performance, while two clusters show a mixture of performance and dropping out of the test. In the following, we present descriptive information about the clusters, first for the “performance” clusters and then for the “drop-out” clusters. A visualization of the clusters can be seen in Figure 2 (academic semester distribution, total scores) and Figure 3 (confidence and correctness per student) with the exact values in Appendix Table 5. Appendix Fig. 4 and Appendix Fig. 5 show cluster to academic semester relation.

**Fig. 4:**
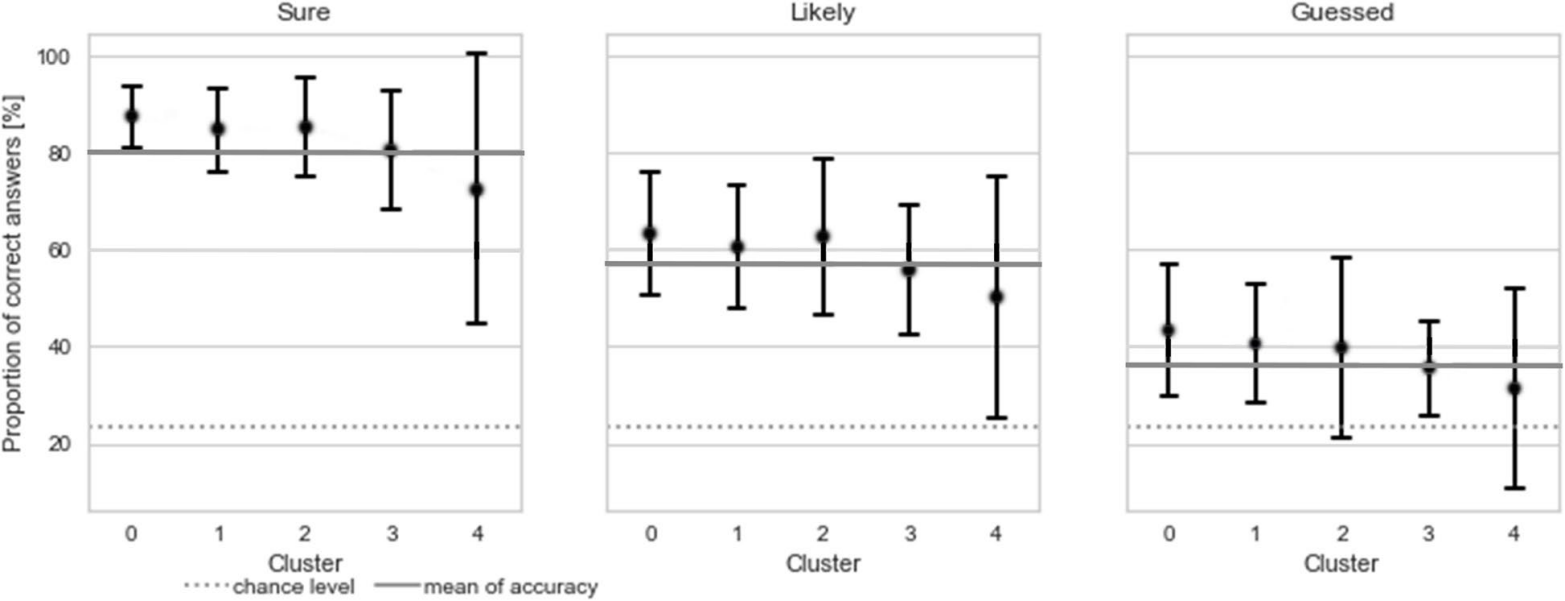
Self-monitoring accuracy per cluster. Each plot shows the mean (± 1 standard deviation) proportion split by confidence relative to the chance level of 23.53 (dashed line) and the total mean per confidence (“sure” = 79.93, “likely” = 56.65, “guessed” = 36.18; gray line)

**Fig. 5:**
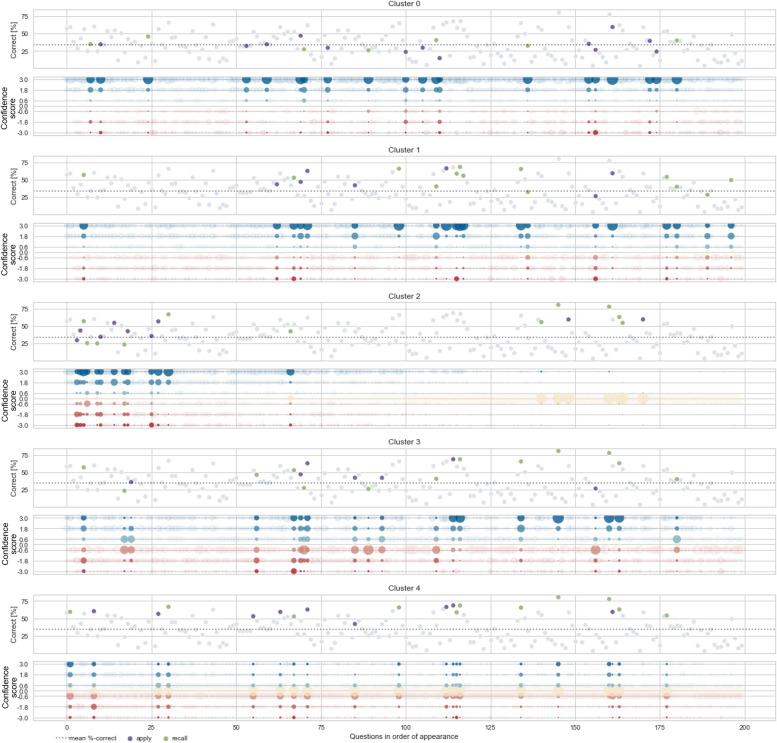
Difficulty index of each question highlighting the 20 most relevant questions per cluster. The questions are ordered by their position in the test with the 20 most relevant questions per cluster highlighted. Each cluster is represented by two graphs. In the upper graph, the relevant questions are colored according to their competence level (“apply” = purple, “recall” = green) and plotted against the difficulty index (correct [%]). In the lower graph blue indicates correctly answered questions and red indicates incorrectly answered questions plotted against the confidence scores. The larger the dots, the more students answered the same way

The “performance” clusters were clusters 0, 1, and 3, as shown in Figure 2. Cluster 0 (*n* = 761) contained students who were mainly close to graduation. These students had the highest mean total score (± standard deviation) of 243.45 (± 48.95); they also had a high proportion of correct answers labeled as “sure” (Figure 2, Appendix Table 7). Cluster 1 (*n* = 1,357) mainly consisted of advanced students, and cluster 3 (*n* = 1,453) consisted of those who were in their early semesters, with a peak at the third semester and were therefore considered beginners. These clusters had mean scores of 135.23 (± 33.87) and 42.60 (± 33.85), respectively.

Cluster 2 (*n* = 384), the smallest cluster, contained mostly students close to graduation who drop out about halfway through the test. These students answered enough questions and remained in the test long enough to be considered serious. Cluster 2 could be classified as late "drop-outs". The mean total score for this cluster was 75.68 (± 41.85). Cluster 4 (*n* = 1,489) contained mainly the lowest scoring students, i.e., students who did not answer enough questions with the accuracy and confidence needed to score higher. They either skipped many questions or provided mostly “guessed” answers if they completed the test at all. The peak was at the first semester (Figure 2). This cluster also included 590 of the 596 students classified as “non-serious”. The mean total score was -18.09 (± 43.25).

Considering their very high number of unanswered questions compared to clusters 0, 1, and 3, clusters 2 and 4 could be regarded as “drop-out” clusters. Although cluster 4 also included a high number of guesses, for ease of distinction, we will refer to these two clusters as “drop-out” clusters for the remainder of the paper.

The main difference between the “performance” clusters and the “drop-out” clusters was the number of unanswered questions. The “performance” clusters averaged 5.94 unanswered questions for cluster 0, 12.67 for cluster 1, and 13.49 for cluster 3, while the two “drop-out” clusters averaged 103.71 unanswered questions for cluster 2 and 97.08 for cluster 4.

The mean results per cluster and confidence level showed that students in the first three clusters (clusters 0, 1, 2) had above-average self-monitoring accuracy at all confidence levels (Figure 4, Appendix Table 6); cluster 3 showed a near-average self-monitoring accuracy and in cluster 4 self-monitoring accuracy was below average with a high standard deviation.

### Classification

The splits consisted of 4,083 observations for training and 1,361 for testing. The splitting was performed 100 times with consecutive training of the boosting algorithm. The model accuracies ranged from 0.855 to 0.899 with a median of 0.876. We used the model with the highest accuracy (0.899) for further analysis. We also obtained the corresponding weighted F1 scores [44], which represents the harmonic mean between weighted precision and weighted recall, which ranged from 0.856 to 0.899. The weighted F1 score of the selected models was also 0.899. Appendix Table 8, Appendix Table 9, and Appendix Fig. 6 show precision and recall, in addition to the aforementioned metrics, for all runs including the selected model.

**Fig. 6:**
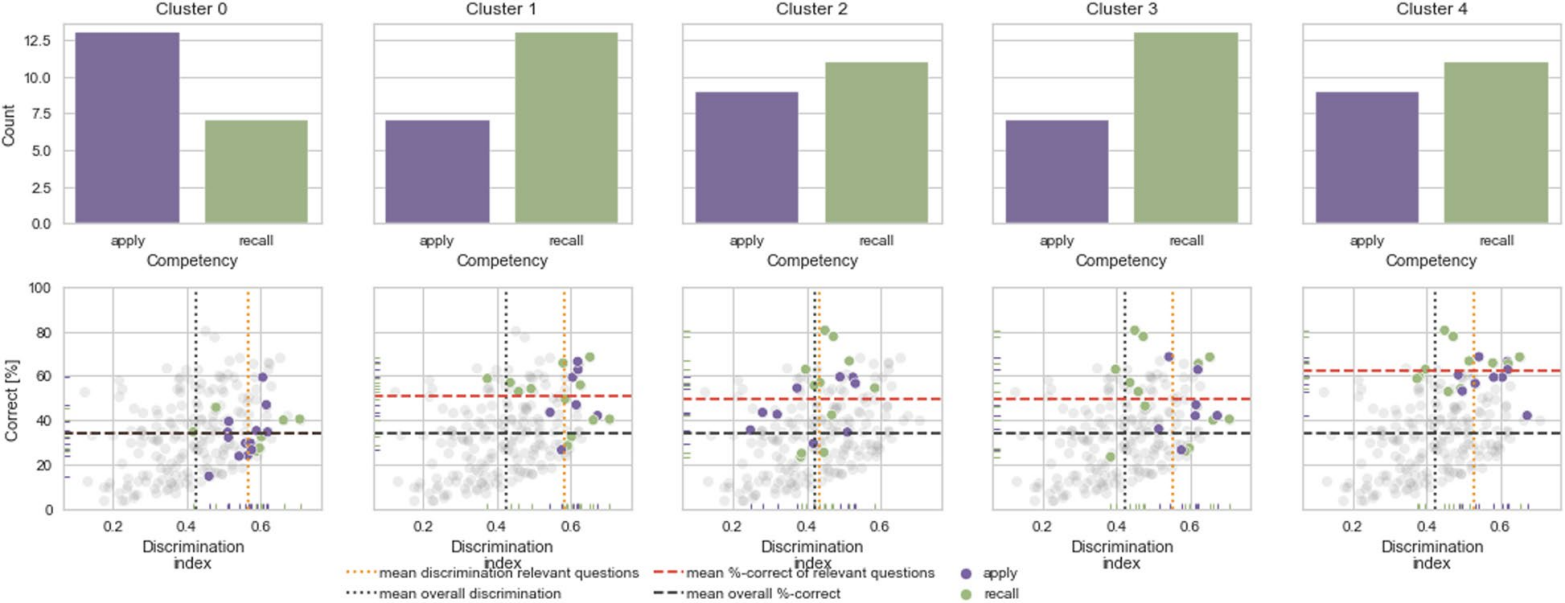
Competence distribution for the 20 most relevant questions for each cluster. The upper plots show the competence level (“apply”, “recall”) of the 20 most relevant questions. In contrast to all other clusters, the competence distribution in cluster 0 is higher for “apply” questions. The lower plots show all questions in terms of their difficulty index (correct [%]) and discrimination index. The relevant questions are highlighted in their respective competence colors

### Explainer

All relevant questions in the “performance” clusters had a discrimination index above 0.3, and almost all of them exceeded the test average (0.4). A large variation was found in the difficulty index. Figure 5 and Figure 6 show the difficulty index, competence level, and discrimination index of the 20 most relevant questions. For clusters 0 and 1, the "relevant questions" were predominantly answered correctly and with confidence “sure”. They differed in terms of difficulty index and competence level. Of the 20 most relevant questions that distinguished cluster 0 from the other clusters, 13 (65%) were of competence level “apply” and only seven (35%) were of competence level “recall”. This is noteworthy because in this PTM run, 45% of the questions were “apply” questions and 55% were “recall” questions. The 20 most relevant questions showed an above-average discrimination index and a slightly lower difficulty index (“more difficult”) than the mean difficulty index of all questions in this test. For the relevant questions in cluster 1, the ratio “apply”:“recall” was reversed (7:13 or 35%:65%). The difficulty index of these questions was on average higher (i.e.,"easier" questions) than the mean difficulty index of all questions in this PTM run, but above average in terms of discrimination index.

The relevant questions in cluster 3 were above average in both difficulty index and discrimination index. However, the response pattern was different: Students answered the “easier” questions correctly with high confidence and “guessed” the difficult questions incorrectly. Here, the ratio “apply”:“recall” equaled that of cluster 1 (35%:65%).

The relevant questions for the two “drop-out” clusters showed different characteristics. For cluster 2, seven (35%) of the relevant questions were “easier”, and were located in the second half of the test. However, cluster 2 students did not answer them. 12 (60%) of the remaining 13 relevant questions were in the first quarter of the test and were mostly answered correctly.

For cluster 4, relevant questions were located throughout the test and were “easier” questions. Cluster 4 students either did not answer or wrongly “guessed” these. The SHAP values for all questions per cluster can be seen in Appendix Table 10.

## Discussion

We explored the response behavior of PTM students to identify groupings of students disregarding test-taking effort, cohorts, and possible curricular differences, but solely based on their performance. To do this, we used a clustering algorithm to detect underlying patterns, followed by a boosting classifier. We then passed the model obtained from classification to an explainer that computed the relevance of each question for each cluster. We selected the 20 most relevant questions per cluster.

These relevant questions should always be considered in conjunction with the corresponding response patterns, as shown by two examples from Figure 5: (1) the students in cluster 3 answered the “easier” relevant questions correctly with high confidence and guessed the “more difficult” relevant questions incorrectly; (2) question 110 is relevant for clusters 0, 1, and 3. The question was answered mostly “sure” and correctly by students in cluster 0, answered “likely” and correctly by students in cluster 1, and guessed incorrectly by students in cluster 3.

### Parameters and performance measurements

The performance measurements for the multiple runs of the clustering and classifier algorithms yielded values in close and reasonable ranges. The accuracy of about 90% suggests that the classifier was able to learn well the assignment of the response patterns to the corresponding clusters. We conclude that the analysis pipeline provides meaningful and interpretable results without hyperparameter tuning.

### Cluster

Our clustering yielded three “performance” clusters and two “drop-out” clusters. Our "performance” clusters divided the course of study into three parts: beginners, advanced, and close to graduation. They reflected the patterns found for example by Cecilio-Fernandes et al. (2016) in their research on progress tests [45]. There, medical students in their early years perform better on simple “recall” questions and medical students closer to graduation perform better on “apply” questions. Our pipeline distinguished cluster 0 students from students of other clusters by the high proportion of correctly answered “apply” questions. Similarly, students in clusters 1 and 3 were identified by their response patterns, which included a higher proportion of “recall” questions and, specifically for cluster 3, a higher number of incorrectly guessed answers. Kämmer et al. (2020) found no differences in self-monitoring accuracy across semesters, with the exception of first-semester students, who were less accurate [9]. Our results support their findings for clusters 0 and 1. The mean self-monitoring accuracy of cluster 3 was slightly lower than the mean self-monitoring accuracy of clusters 0 and 1 (Figure 4) and the mean self-monitoring accuracy reported by Kämmer et al. (2020) [9]. However, cluster 3 included also students beyond their first semester.

The following was found on the “drop-out” clusters: the pattern of responded questions in cluster 2 was similar to that in clusters 0 and 1, which suggests that most of the students would likely have achieved scores comparable to those of clusters 0 and 1 had they completed the test. Additionally, students in clusters 0, 1, and 2 showed similar self-monitoring accuracy in confidence (Figure 4). Since traditional numerical considerations underestimate student knowledge in cluster 2, our clusters add a new perspective to this formative assessment.

Cluster 4 contained almost all students whose participation was considered “non-serious” and the first semester students who mainly guessed, which amounts for more than half of the first semester students. Proposing two subgroups in this cluster is supported by cluster 4’s high standard deviation in self-monitoring accuracy. It would be questionable to refer to this cluster as “drop-outs”. Wang et al. (2021) identified four latent states in students' progress test scores: Novice, Advanced Beginner I, Advanced Beginner II, and Competent [19]. Our “performance” clusters resemble their states. Our first semester students in cluster 4 might resemble their "novice" state. Unlike the PTM, their progress test contributes to students' grades as well as to decisions about progression in the course of study [19]. Our "drop-out" clusters reflect the purely formative nature of the PTM.

### Order of questions

There will always be students who just answer the first half of the questions and therefore fall in cluster 2. We wonder if most of the relevant questions identified by the explainer algorithm for the “performance” clusters will inevitably be located after the first quarter of the test, regardless of their content.

### Inclusion in PTM feedback

Our clusters provide an addition to traditional cohort-based numerical feedback. Grouping similarly performing students across cohorts and universities can help create a profile that focuses on their strengths and weaknesses. Such a personalized analysis is a known factor for effective feedback (e.g. [46] and references therein) and is requested by PTM students, as shown by PTM surveys [15, 16]. For example, students can visually compare their response patterns with the response patterns of all clusters and thus get a better overview of their current level of knowledge. Students in cluster 2 gain a better estimation of their performance on the first half of the test than by using only standard numerical feedback and averaged scores.

### Limitations

An imbalance in the data originated from the fact that the largest university administers the test every term and admits new students twice a year and smaller universities have an uneven distribution of participating students and not all offer the PTM every term. Since we want to represent reality, we consider it necessary to preserve the original dataset structure.

In the original data, each student’s answer was represented by an identifier consisting of the confidence in the given answer and its correctness. For clustering, we translated these categories into numerical values, knowing that this transition may have an impact on our clustering results. Our scoring assignment was based on a mathematical background. We find the resulting clusters reasonable and interpretable for our purpose.

### Future research

In this study, we only included the students’ scores in our analysis. Future research could include other parameters to adjust for certain distractions. For example, one feature that could be included is the amount of time a student spends on a question. This could help to find new cluster indicators and possibly give us even more insight into the differences between certain groups of students.

Longitudinal analysis of both students and relevant questions is also of great interest. A student's retention in a cluster, or transition from one cluster to another during the course of study, could provide information about students' knowledge gains and infer developmental patterns [19], which would be consistent with the goal to provide feedback to address students' future development as described in the literature (e.g. [47–49] and references therein). For example, early indicators could be used to identify individuals in need of support.

To investigate whether the order of questions in a test has an effect on identifying the relevant questions for the clusters, we propose the following approaches for a next test:

-  → offer the same questions in the same order
-  → offer the same questions in a different order
-  → of a former test, replace the non-relevant questions and keep the position of the relevant questions
-  → place all relevant questions of the “performance” clusters of a former test at the beginning of the test

-  → offer a shorter test consisting of the relevant question of the “performance” cluster of a former test

## Conclusion

We found three different “performance” clusters and two “drop-out” clusters.

Students in the clusters differed in terms of stage of study, knowledge of easier and more difficult questions, and confidence in their answers and accuracy of this self-monitoring. The “performance” clusters divide the course of study into beginners, advanced and close to graduation. Students in one “performance” cluster are distinguished by their high proportion of correctly answered relevant “apply” questions. Students in the other two “performance” clusters are identified by their higher proportion of relevant “recall” questions, for example. The analysis of a “drop-out” cluster suggests that most students of that cluster had the chance to be in the “performance” clusters had they completed the test.

## Supplementary Information

**Additional file 1:** **Appendix Table 1.** Students per semester.** Appendix Table 2.** Students per university. **Appendix Figure 1.** Distribution of discrimination indices of questions from one ‘Progress Test Medizin’ run. The dotted line at 0.3 shows the well discriminating question threshold. **Appendix Figure 2.** Test scores. Percentages of correct answers per students grouped by semester. Overall, 5,444 students from 8 universities in Germany and Austria are shown. Each dot represents the share of correct answers of a single participation. **Appendix Table 3.** Overall accuracy of confidence. **Appendix Figure 3.** Distortion score elbow for k-means clustering. Mathematically determining the optimal number of clusters k for applying k-means on the PTM data from winter term 2020. Possible k ranges were set between 1 and 29. For each potential k (x-axis), the distortion score (left y-axis) for received clustering and the time it needs to fit in seconds (right y-axis) are shown in blue and in green, respectively. The optimal k based on this run was 5. **Appendix Table 4.** Descriptive statistics of the Calinsky-Harabasz score from 200 k-means runs. The model with the maximum Calinsky-Harabasz score was kept as final model. **Appendix Figure 4.** Academic semester distribution per cluster. For each academic semester, the distributions of the students in the different clusters are shown in percent. Same colors sum up to 100. For example, ~46 % of students from academic semester 7 are in cluster 1. Raw count distribution can be seen in Figure 3. (Appendix Figure 5 shows the same percent, but ordered by academic semester and colored by cluster). **Appendix Figure 5.** Cluster distribution per academic semester. For each academic semester, the distributions of the cluster association for each academic semester is shown in percent. Each academic semester-group sums up to 100. For example, ~46 % of students from semester 7 are in cluster 1. (Appendix Figure 4 shows the same percent, but ordered by cluster and colored by academic semester). **Appendix Table 5.** Number of observations and descriptive statistics of total score per cluster. **Appendix Table 6.** Self-monitoring accuracy by cluster. **Appendix Table 7.** Descriptive statistics of scores per cluster. **Appendix Table 8.** Descriptive statistics of performance measures from 100 XGBoost runs. **Appendix Figure 6.** Visualization of performance measures for all 100 XGBoost runs. **Appendix Table 9.** Performance measures for test data (*N*=1,361) with the final classifier. **Appendix Table 10.** Absolute SHAP-value of each question for each cluster.

## Data Availability

The datasets generated during and/or analyzed during the current study are not publicly available for data security reasons but are available from the corresponding author on reasonable request and after approval of the Progress Test cooperation partners and an extended ethical approval.

## Abbreviations

PTM: Progress Test Medizin
CHS: Calinski-Harabasz score
