# Supplementary material for: Discovering unknown response patterns in progress test data to improve the estimation of student performance
Source: BMC Med Educ. 2023 Mar 29;23:193. doi: 10.1186/s12909-023-04172-w (PMC10053036; doi:10.1186/s12909-023-04172-w)
Supplement: Supplementary file 1 — Additional file 1: Appendix Table 1. Students per semester. Appendix Table 2. Students per university. Appendix Figure 1. Distribution of discrimination indices of questions from one ‘Progress Test Medizin’ run. The dotted line at 0.3 shows the well discriminating question threshold. Appendix Figure 2. Test scores. Percentages of correct answers per students grouped by semester. Overall, 5,444 students from 8 universities in Germany and Austria are shown. Each dot represents the share of correct answers of a single participation. Appendix Table 3. Overall accuracy of confidence. Appendix Figure 3. Distortion score elbow for k-means clustering. Mathematically determining the optimal number of clusters k for applying k-means on the PTM data from winter term 2020. Possible k ranges were set between 1 and 29. For each potential k (x-axis), the distortion score (left y-axis) for received clustering and the time it needs to fit in seconds (right y-axis) are shown in blue and in green, respectively. The optimal k based on this run was 5. Appendix Table 4. Descriptive statistics of the Calinsky-Harabasz score from 200 k-means runs. The model with the maximum Calinsky-Harabasz score was kept as final model. Appendix Figure 4. Academic semester distribution per cluster. For each academic semester, the distributions of the students in the different clusters are shown in percent. Same colors sum up to 100. For example, ~46 % of students from academic semester 7 are in cluster 1. Raw count distribution can be seen in Figure 3. (Appendix Figure 5 shows the same percent, but ordered by academic semester and colored by cluster). Appendix Figure 5. Cluster distribution per academic semester. For each academic semester, the distributions of the cluster association for each academic semester is shown in percent. Each academic semester-group sums up to 100. For example, ~46 % of students from semester 7 are in cluster 1. (Appendix Figure 4 shows the same percent, but ordered by cluster [file 12909_2023_4172_MOESM1_ESM.pdf]

Supplementary Material

Discovering unknown response patterns in progress test data to improve the estimation of student performance

Miriam Sieg[1,2], Iván Roselló Atanet[1], Mihaela Todorova Tomova[3], Uwe Schoeneberg[2], Victoria Sehy[1], Patrick Mäder[3,4], Maren März[1*]

1. Research Resource Identifiers

Python:	Python Version 3.8.3, RRID:SCR_008394
k-means:	scikit-learn, RRID:SCR_002577
XGBoost:	XGBoost, RRID:SCR_021361
TreeSHAP:	SHapley Additive ExPlanations, RRID:SCR_021362

2. Additional tables and figures

Appendix tables content

Appendix Table 1 **Students per semester** 2
Appendix Table 2 **Students per university** 2
Appendix Table 3 **Overall accuracy of confidence** 3
Appendix Table 4 **Descriptive statistics of the Calinsky-Harabasz score from 200 *k-means* runs**. The model with the maximum Calinsky-Harabasz score was kept as final model. 3
Appendix Table 5 **Number of observations and descriptive statistics of total score per cluster.** 5
Appendix Table 6 **Self-monitoring accuracy by cluster** 5
Appendix Table 7 **Descriptive statistics of scores per cluster** 6
Appendix Table 8 **Descriptive statistics of performance measures from 100 XGBoost runs** 7
Appendix Table 9 **Performance measures for test data (N=1,361) with the final classifier** 7
Appendix Table 10 **Absolute SHAP-value of each question for each cluster** 8

Appendix figures content

Appendix Figure 1 **Distribution of discrimination indices of questions from one 'Progress Test Medizin' run.** The dotted line at *0.3* shows the well discriminating question threshold. 2
Appendix Figure 2 **Test scores.** Percentages of correct answers per students grouped by semester. Overall, 5,444 students from 8 universities in Germany and Austria are shown. Each dot represents the share of correct answers of a single participation. 2
Appendix Figure 3 **Distortion score elbow for *k-means* clustering.** Mathematically determining the optimal number of clusters *k* for applying *k-means* on the PTM data from winter term 2020. Possible *k* ranges were set between 1 and 29. For each potential *k* (x-axis), the distortion score (left y-axis) for received clustering and the time it needs to fit in seconds (right y-axis) are shown in blue and in green, respectively. The optimal *k* based on this run was 5. 3
Appendix Figure 4 **Academic semester distribution per cluster.** For each academic semester, the distributions of the students in the different clusters are shown in percent. Same colors sum up to 100. For example, ~46 % of students from academic semester 7 are in cluster 1. Raw count distribution can be seen in Figure 3. (Appendix Figure 5 shows the same percent, but ordered by academic semester and colored by cluster) 4
Appendix Figure 5 **Cluster distribution per academic semester.** For each academic semester, the distributions of the cluster association for each academic semester is shown in percent. Each academic semester-group sums up to 100. For example, ~46 % of students from semester 7 are in cluster 1. (Appendix Figure 4 shows the same percent, but ordered by cluster and colored by academic semester) 4
Appendix Figure 6: **Visualization of performance measures for all 100 XGBoost runs** 7

Appendix Table 1 **Students per semester**

1	2	3	4	5	6	7	8	9	10
622	475	942	423	646	383	545	391	645	372

Appendix Table 2 **Students per university**

1	2	3	4	5	6	7	8
3,077	526	506	403	308	287	185	152

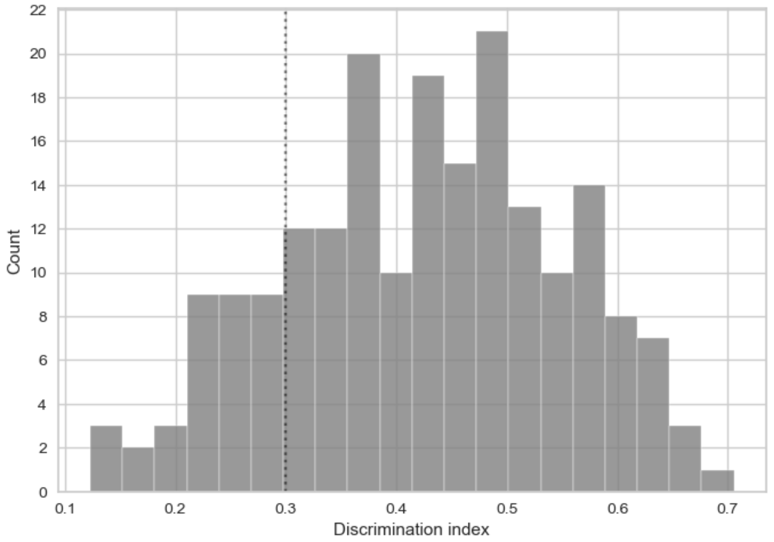

Appendix Figure 1 **Distribution of discrimination indices of questions from one 'Progress Test Medizin' run.** The dotted line at *0.3* shows the well discriminating question threshold.

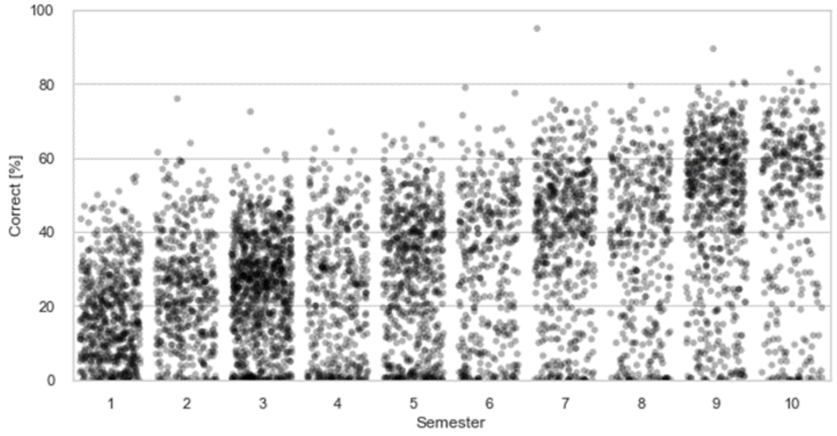

Appendix Figure 2 **Test scores.** Percentages of correct answers per students grouped by semester. Overall, 5,444 students from 8 universities in Germany and Austria are shown. Each dot represents the share of correct answers of a single participation.

Appendix Table 3 **Overall accuracy of confidence**

	**Average of correct answers[%]**	**Difference to average of preceding confidence**
	0	
**chance**	23.53	23.53
**guessed**	36.18	12.65
**likely**	56.65	20.47
**sure**	79.93	23.28

CLUSTERING

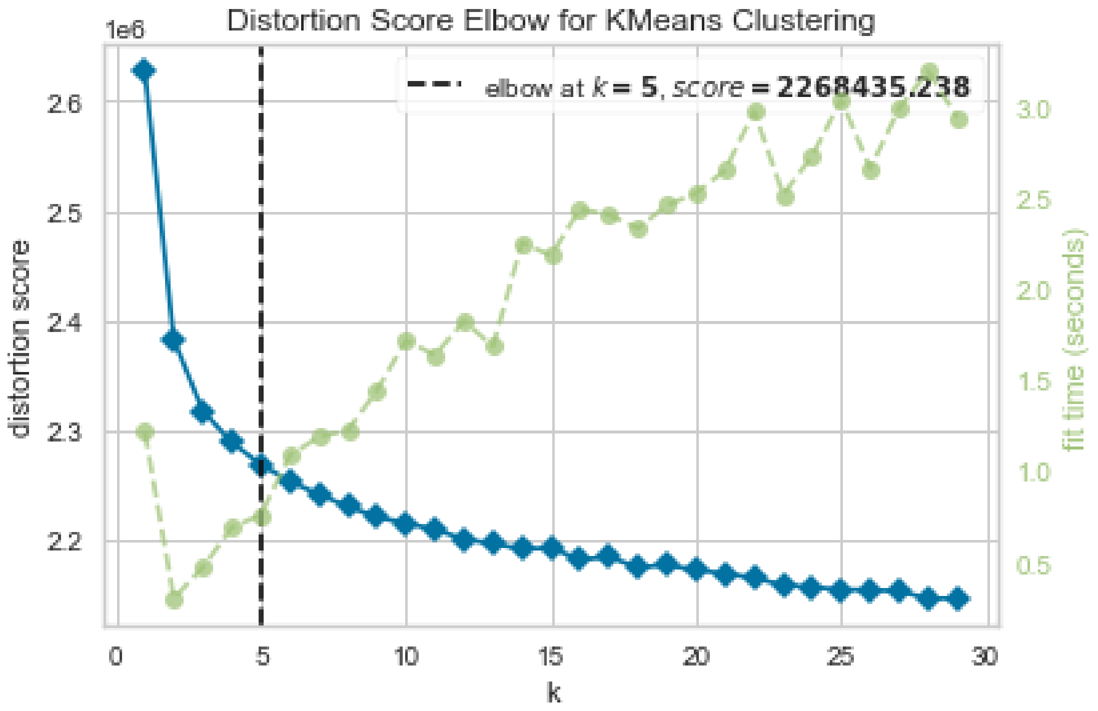

Appendix Figure 3 **Distortion score elbow for *k-means* clustering.** Mathematically determining the optimal number of clusters *k* for applying *k-means* on the PTM data from winter term 2020. Possible *k* ranges were set between 1 and 29. For each potential *k* (x-axis), the distortion score (left y-axis) for received clustering and the time it needs to fit in seconds (right y-axis) are shown in blue and in green, respectively. The optimal *k* based on this run was 5.

Appendix Table 4 **Descriptive statistics of the Calinsky-Harabasz score from 200 *k-means* runs**. The model with the maximum Calinsky-Harabasz score was kept as final model.

**Mean**	**Std**	**Minimum**	**25%**	**50%**	**75%**	**Maximum**
214.07	1.20	212.55	212.61	215.04	215.05	215.08

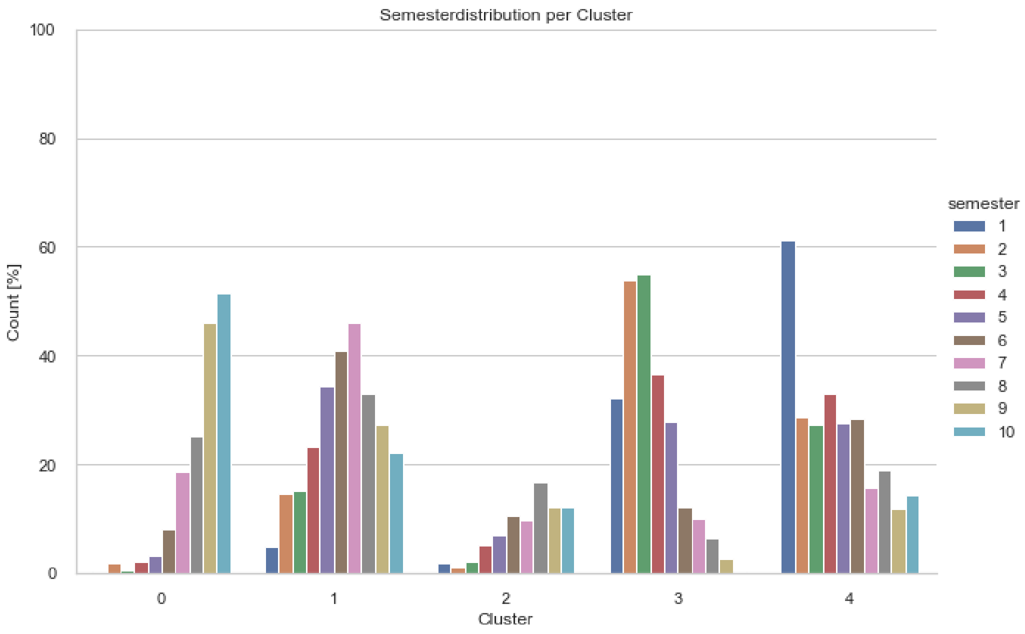

Appendix Figure 4 **Academic semester distribution per cluster.** For each academic semester, the distributions of the students in the different clusters are shown in percent. Same colors sum up to 100. For example, ~46 % of students from academic semester 7 are in cluster 1. Raw count distribution can be seen in Figure 3. (Appendix Figure 5 shows the same percent, but ordered by academic semester and colored by cluster)

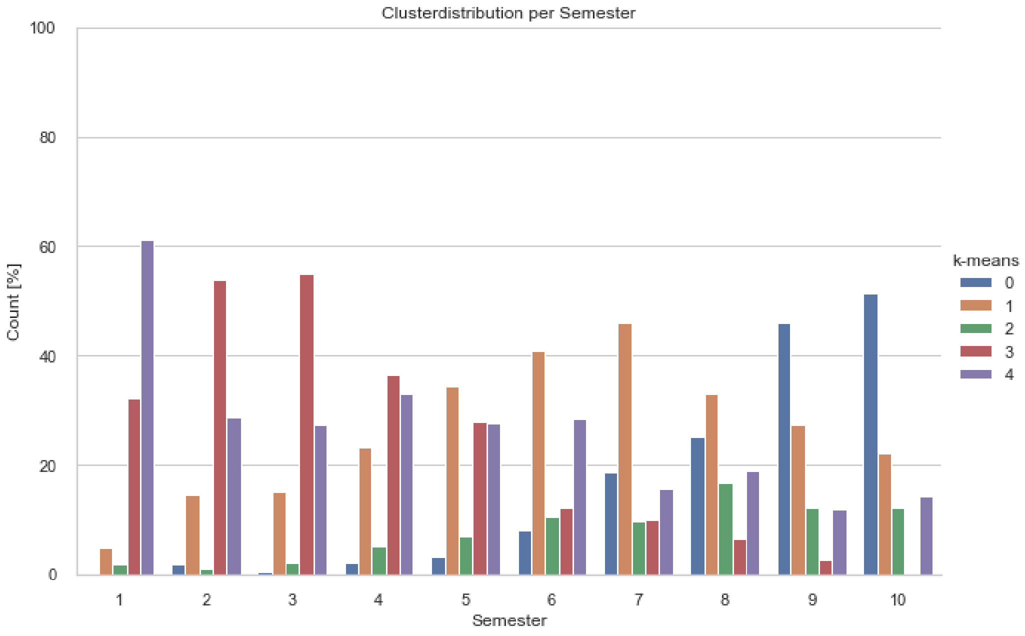

Appendix Figure 5 **Cluster distribution per academic semester.** For each academic semester, the distributions of the cluster association for each academic semester is shown in percent. Each academic semester-group sums up to 100. For example, ~46 % of students from semester 7 are in

cluster 1. (Appendix Figure 4 shows the same percent, but ordered by cluster and colored by academic semester)

Appendix Table 5 **Number of observations and descriptive statistics of total score per cluster.**

Cluster	Count	Mean	Std	Minimum	0.25	0.5	0.75	Maximum
**0**	761	243.45	48.95	146.4	208.8	232.8	271.2	543
**1**	1,357	135.23	33.87	44.4	109.2	135.6	160.8	242.4
**2**	384	75.68	41.85	-52.8	46.05	66	99.6	209.4
**3**	1,453	42.6	33.85	-134.4	20.4	45	67.2	129.6
**4**	1,489	-18.09	43.25	-319.8	-33.6	-3	5.4	58.8

Appendix Table 6 **Self-monitoring accuracy by cluster**

Cluster	Sure [%]		Likely [%]		Guessed [%]	
	Mean	Std	Mean	Std	Mean	Std
**0**	87.56	6.41	63.38	12.9	43.36	13.56
**1**	84.89	8.69	60.59	12.67	40.63	12.19
**2**	85.34	10.38	62.64	16.09	39.83	18.54
**3**	80.57	12.14	55.69	13.43	35.52	9.88
**4**	72.5	27.92	50.15	24.92	31.34	20.73

Appendix Table 7 **Descriptive statistics of scores per cluster**

Score (Mapping)	Cluster	min	25%	50%	75%	max
**-3** (incorrect, sure)	0	0	6	11	18	71
	1	0	4	8	15	91
	2	0	1	4	7	108
	3	0	2	5	11	112
	4	0	0	1	3	152
**-1.8** (incorrect, likely)	0	0	13	20	27	59
	1	0	16	24	33	75
	2	0	6	11	17	110
	3	0	14	25	37	111
	4	0	1	5	16	154
**-0.6** (incorrect, guessed)	0	0	11	20	29	56
	1	0	22	37	49	88
	2	0	4	10	22	116
	3	0	42	64	81	125
	4	0	1	17	102	170
**0** (no answer)	0	0	0	0	1	82
	1	0	0	0	4	143
	2	0	93	107	138	177
	3	0	0	0	1	167
	4	0	0	126	188	199
**0.6** (correct, guessed)	0	0	7	15	23	65
	1	0	14	24	35	91
	2	0	2	8	17	65
	3	0	20	33	45	104
	4	0	0	9	42	130
**1.8** (correct, likely)	0	0	25	35	48	150
	1	0	26	37	49	143
	2	0	12	19	28	71
	3	0	22	30	39	117
	4	0	1	6	16	80
**3** (correct, sure)	0	10	68	84	101	188
	1	0	37	49	62	127
	2	0	15	25	38	88
	3	0	15	24	34	99
	4	0	1	3	8	81

CLASSIFICATION

Appendix Table 8 **Descriptive statistics of performance measures from 100 XGBoost runs**

Performance Measure	Mean	Std	Minimum	0.25	0.5	0.75	Maximum
**Precision**	0.88	0.008	0.859	0.875	0.879	0.885	0.901
**Recall**	0.876	0.008	0.855	0.871	0.876	0.882	0.899
**F1 score**	0.877	0.008	0.856	0.872	0.877	0.882	0.899
**Accuracy**	0.876	0.008	0.855	0.871	0.876	0.882	0.899

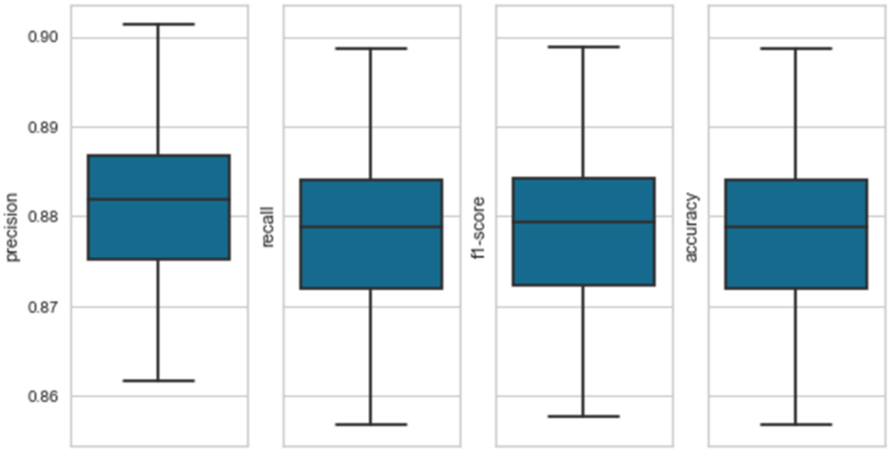

Appendix Figure 6: **Visualization of performance measures for all 100 XGBoost runs**

Appendix Table 9 **Performance measures for test data (N=1,361) with the final classifier**

Cluster	Precision	Recall	F1-Score
0	0.94	0.858	0.897
1	0.862	0.893	0.877
2	0.859	0.839	0.849
3	0.864	0.911	0.887
4	0.968	0.927	0.947
**Weighted Average**	**0.901**	**0.899**	**0.899**

EXPLAINER

Appendix Table 10 **Absolute SHAP-value of each question for each cluster**

	**Absolute SHAP-Values**				
**Question**	Cluster 0	Cluster 1	Cluster 2	Cluster 3	Cluster 4
**0**	0.001	0.003	0.016	0.004	0.009
**1**	0.002	0.031	0.047	0.029	0.149
**2**	0.069	0.011	0.052	0.027	0.042
**3**	0.075	0.039	0.073	0.005	0.037
**4**	0.041	0.019	0.089	0.015	0.041
**5**	0.009	0.112	0.208	0.204	0.081
**6**	0.062	0.036	0.094	0.009	0.014
**7**	0.125	0.016	0.023	0.01	0.015
**8**	0.01	0.013	0.021	0.015	0.132
**9**	0.029	0.008	0.102	0.02	0.033
**10**	0.284	0.032	0.09	0.024	0.042
**11**	0	0.009	0.061	0.001	0.027
**12**	0.081	0.016	0.062	0.04	0.101
**13**	0.003	0.014	0.035	0.01	0.051
**14**	0.01	0.002	0.076	0.003	0.033
**15**	0.006	0.002	0.009	0.008	0.001
**16**	0	0.028	0.029	0.011	0.016
**17**	0.014	0.008	0.12	0.116	0.012
**18**	0.003	0.002	0.128	0.007	0.032
**19**	0.005	0.021	0.037	0.135	0.021
**20**	0.004	0.029	0.049	0.011	0.048
**21**	0.007	0.003	0.005	0.002	0.006
**22**	0.004	0.003	0.014	0.011	0.003
**23**	0.007	0.002	0.008	0.004	0.004
**24**	0.114	0.036	0.011	0.024	0.04
**25**	0	0.01	0.097	0.005	0.012
**26**	0.018	0.007	0.011	0.002	0.001
**27**	0.067	0.013	0.087	0.033	0.17
**28**	0.006	0.003	0.038	0.008	0.007
**29**	0.003	0.015	0.013	0.03	0.014
**30**	0.01	0.037	0.187	0.045	0.253
**31**	0.103	0.015	0.026	0.012	0.035
**32**	0.003	0.005	0.012	0.006	0.004
**33**	0.008	0.003	0.022	0.007	0.018
**34**	0.001	0.005	0.048	0.004	0.032
**35**	0.039	0.003	0.019	0.004	0.087

**36**	0.057	0.012	0.007	0.005	0.002
**37**	0.003	0.006	0.011	0.01	0.001
**38**	0	0.035	0.033	0.036	0.013
**39**	0.036	0.008	0.024	0.056	0.047
**40**	0.021	0.022	0.019	0.015	0.013
**41**	0.008	0.005	0.005	0.007	0.005
**42**	0.011	0.01	0.01	0.005	0.004
**43**	0.009	0.01	0.004	0.014	0.009
**44**	0.016	0.008	0.008	0.023	0.012
**45**	0.002	0.011	0.017	0.004	0.002
**46**	0.005	0.009	0.019	0.003	0.021
**47**	0	0.003	0.003	0.004	0.003
**48**	0.02	0.006	0.067	0.013	0.047
**49**	0.009	0.006	0.038	0.004	0.075
**50**	0.101	0.005	0.026	0.057	0.023
**51**	0.035	0.027	0.022	0.006	0.004
**52**	0.002	0.011	0.015	0.013	0.048
**53**	0.154	0.046	0.025	0.046	0.003
**54**	0.033	0.016	0.02	0.018	0.013
**55**	0.004	0.018	0.005	0.011	0.142
**56**	0.075	0.059	0.049	0.177	0.015
**57**	0.007	0.015	0.011	0.004	0.024
**58**	0	0.021	0.006	0.009	0.008
**59**	0.227	0.051	0.027	0.083	0.056
**60**	0.013	0.004	0.002	0.03	0.006
**61**	0.019	0.012	0.001	0.007	0.071
**62**	0.044	0.078	0.018	0.039	0.027
**63**	0	0.008	0.039	0.014	0.188
**64**	0.025	0.01	0.068	0.015	0.026
**65**	0.024	0.002	0.008	0.039	0.042
**66**	0.045	0.065	0.078	0.058	0.044
**67**	0.024	0.086	0.069	0.142	0.234
**68**	0.009	0.014	0.006	0.032	0.014
**69**	0.149	0.096	0.036	0.128	0.056
**70**	0.342	0.054	0.009	0.121	0.013
**71**	0.009	0.173	0.046	0.096	0.33
**72**	0.062	0.013	0.017	0.04	0.003
**73**	0.003	0.005	0.004	0.029	0
**74**	0.003	0.003	0.002	0.026	0.057
**75**	0.023	0.007	0.001	0.01	0.005
**76**	0.001	0.002	0.001	0.009	0.024

**77**	0.268	0.028	0.004	0.033	0
**78**	0.004	0.007	0.011	0.002	0.002
**79**	0.001	0.001	0.002	0.007	0.001
**80**	0.031	0.01	0.012	0.015	0.1
**81**	0.085	0.012	0	0.021	0.005
**82**	0.021	0.059	0.007	0.068	0.088
**83**	0.051	0.009	0.004	0.005	0.038
**84**	0.084	0.014	0.004	0.094	0.002
**85**	0.016	0.122	0.006	0.223	0.2
**86**	0.032	0.016	0.019	0.005	0.065
**87**	0.008	0.009	0.003	0.002	0.019
**88**	0.007	0.012	0.001	0.018	0.001
**89**	0.395	0.059	0.002	0.113	0.001
**90**	0.01	0.012	0.017	0.004	0.002
**91**	0.01	0.004	0	0.002	0.002
**92**	0.035	0.036	0.03	0.008	0.003
**93**	0.046	0.033	0.004	0.183	0.006
**94**	0.012	0.062	0	0.027	0.011
**95**	0.023	0.002	0.002	0.008	0
**96**	0.001	0.007	0.002	0.006	0.014
**97**	0.035	0.009	0.028	0.015	0.01
**98**	0.003	0.228	0.022	0.036	0.196
**99**	0.022	0.009	0	0.011	0.005
**100**	0.163	0.019	0.008	0.029	0.003
**101**	0.006	0.006	0.003	0.001	0.002
**102**	0.036	0.042	0.003	0.008	0.002
**103**	0.009	0.014	0	0.005	0.003
**104**	0.01	0.025	0.001	0.007	0.006
**105**	0.167	0.032	0.011	0.027	0
**106**	0.005	0.016	0	0.002	0.001
**107**	0.013	0.006	0.005	0.009	0.002
**108**	0.02	0.016	0.07	0.02	0.109
**109**	0.321	0.342	0.001	0.223	0.035
**110**	0.13	0.029	0.002	0.046	0.001
**111**	0.005	0.022	0.057	0.045	0.049
**112**	0.003	0.086	0.011	0.089	0.525
**113**	0.007	0.006	0	0.002	0
**114**	0.001	0.047	0.021	0.109	0.277
**115**	0.002	0.189	0	0.016	0.13
**116**	0.003	0.512	0.009	0.321	0.673
**117**	0.002	0.164	0	0.07	0.03

**118**	0.002	0.033	0.005	0.027	0.07
**119**	0.02	0.009	0.006	0.019	0
**120**	0.086	0.024	0	0.011	0.001
**121**	0.094	0.011	0	0.033	0.005
**122**	0.012	0.05	0.071	0.012	0.007
**123**	0.002	0.001	0.002	0.002	0.001
**124**	0.009	0.017	0.002	0.012	0.032
**125**	0.017	0.018	0.01	0.003	0
**126**	0.021	0.022	0	0.01	0.012
**127**	0.001	0.004	0	0.003	0
**128**	0.04	0.011	0.006	0.009	0
**129**	0.007	0.004	0.003	0.005	0.005
**130**	0.11	0.045	0.006	0.004	0.001
**131**	0.114	0.012	0.003	0.043	0
**132**	0.003	0.004	0.007	0.011	0
**133**	0.015	0.01	0.066	0.021	0.043
**134**	0.004	0.387	0.028	0.117	0.36
**135**	0.008	0.047	0.035	0.016	0.011
**136**	0.283	0.09	0.008	0.045	0.02
**137**	0.007	0.006	0.004	0.005	0.001
**138**	0.005	0.013	0.005	0.004	0
**139**	0.001	0.048	0.006	0.042	0.062
**140**	0.022	0.06	0.082	0.04	0.036
**141**	0.017	0.008	0.006	0.004	0.004
**142**	0.003	0.005	0.009	0.009	0.002
**143**	0.082	0.049	0.001	0.035	0.001
**144**	0.001	0.011	0.001	0.005	0
**145**	0	0.015	0.772	0.456	0.142
**146**	0.002	0.03	0.017	0.018	0.006
**147**	0.002	0.001	0	0.003	0.002
**148**	0.025	0.03	0.377	0.01	0.066
**149**	0.023	0.021	0.007	0.007	0.102
**150**	0.009	0.021	0	0.001	0
**151**	0.002	0.018	0.04	0.005	0.007
**152**	0.072	0.039	0.001	0.014	0.003
**153**	0.004	0.014	0	0.006	0.016
**154**	0.12	0.064	0	0.027	0.004
**155**	0.046	0.022	0.024	0.003	0.002
**156**	0.116	0.074	0	0.108	0
**157**	0.012	0.005	0	0.03	0.007
**158**	0.002	0.001	0.004	0.005	0.002

**159**	0.015	0.019	0	0	0.001
**160**	0.004	0.019	0.751	0.174	0.149
**161**	0.208	0.419	0.054	0.063	0.255
**162**	0.024	0.009	0	0.058	0
**163**	0.008	0.009	0.569	0.12	0.22
**164**	0.006	0.033	0.094	0.026	0.1
**165**	0.017	0.005	0	0.004	0.003
**166**	0.01	0.014	0	0.017	0.03
**167**	0.009	0.008	0.019	0.033	0.002
**168**	0.011	0.005	0	0.009	0.006
**169**	0.012	0.003	0.014	0.005	0
**170**	0.013	0.006	0.3	0.047	0.023
**171**	0	0.012	0.012	0.012	0.042
**172**	0.205	0.029	0	0.064	0.022
**173**	0.035	0.006	0	0.004	0
**174**	0.44	0.03	0	0.018	0.001
**175**	0.011	0.003	0	0.002	0.002
**176**	0	0.012	0.019	0.071	0.034
**177**	0.001	0.085	0	0.031	0.167
**178**	0.046	0.047	0	0.037	0.008
**179**	0.003	0.009	0.002	0.015	0.003
**180**	0.304	0.093	0.042	0.244	0.001
**181**	0.013	0.004	0.005	0.016	0.014
**182**	0	0.002	0	0.009	0.004
**183**	0.006	0.006	0	0.032	0.002
**184**	0.004	0.03	0	0.008	0.015
**185**	0.007	0.004	0	0.014	0.03
**186**	0	0.002	0.01	0.002	0.006
**187**	0.005	0.003	0	0.014	0.007
**188**	0.002	0.001	0	0.003	0
**189**	0.029	0.083	0	0.095	0.001
**190**	0.024	0.052	0	0.067	0.036
**191**	0.02	0.022	0	0.002	0.006
**192**	0.011	0.014	0	0.002	0.002
**193**	0.013	0.02	0.012	0.083	0.006
**194**	0.011	0.012	0.002	0.005	0.002
**195**	0.002	0.002	0	0.002	0.002
**196**	0.015	0.076	0.004	0.021	0.056
**197**	0.024	0.013	0	0.015	0.009
**198**	0.013	0.001	0	0.001	0
**199**	0.027	0.026	0	0.002	0.001
